# Prognostic factors analysis of patients with oral cavity squamous cell carcinoma

**DOI:** 10.1016/j.bjorl.2026.101805

**Published:** 2026-03-26

**Authors:** Gustavo Mercuri, Isabella Grieger, Daniel Naves de Araújo, Fabio Lau, Carlos Takahiro Chone

**Affiliations:** Universidade Estadual de Campinas (UNICAMP), Departamento de Otorrinolaringologia e Cirurgia de Cabeça e Pescoço, Campinas, SP, Brazil

**Keywords:** Survival analysis, Oral cavity squamous cell carcinoma, Oral cavity neoplasm, Oral cancer

## Abstract

**Objective:**

This study aimed to analyze prognostic factors associated with survival and recurrence in patients with Oral Cavity Squamous Cell Carcinoma (OCSCC) treated in São Paulo, Brazil. The investigation focused on evaluating the influence of clinical stage, treatment modality, age, and sex on overall and recurrence-free survival in a large cohort derived from a public cancer registry.

**Study design:**

Single-center retrospective observational study with a cross-sectional and quantitative design.

**Setting:**

São Paulo, Brazil.

**Methods:**

A retrospective analysis was conducted using data from 10,122 patients diagnosed with OCSCC between 2004 and 2014, obtained from the Oncocenter Foundation of São Paulo (FOSP) database. The primary endpoint was Overall Survival (OS), and secondary endpoints included Recurrence-Free Survival (RFS), Disease-Free Survival (DFS), and Local Recurrence-Free Survival (LRFS). Prognostic factors were assessed through univariable and multivariable Cox proportional hazards models. Kaplan-Meier curves were used to estimate survival, and comparisons were made using the log-rank test.

**Results:**

The mean follow-up time was 39.37 ± 37.93 months. Age ranged from 11- to 104-years, with a mean age of 60.2 ± 12.6 years at diagnosis, and 78.3% were male. Most patients (61.97%) were diagnosed at an advanced stage (III–IV). Advanced stage and non-surgical treatment were independently associated with worse OS (HR = 4.253, 95% CI 3.883–4.659; 2.546, 95% CI 2.381–2.722, respectively). The 5-year overall survival rate was 50.3%, with a mean survival interval of 6.70 ± 0.06 years after diagnosis. Elderly patients and men had higher recurrence and mortality risks, whereas early-stage disease and surgical treatment were associated with better outcomes.

**Conclusion:**

Advanced clinical stage and non-surgical treatment were the strongest independent prognostic factors for decreased survival in OSCC. These findings underscore the importance of early detection and surgical management, as well as the need for public health strategies to reduce diagnostic and treatment delays in Brazil.

**Level of evidence:**

2B.

## Highlights

• Surgical treatment improves survival and recurrence-free outcomes in OSCC.
• Early-stage tumors have lower recurrence rates and better prognosis.
• Advanced-stage diagnosis significantly worsens outcomes.
• Large cohort study with over 10,000 patients from São Paulo, Brazil.

## Introduction

Squamous Cell Carcinoma (SCC) represents approximately 90%–95% of malignant neoplasms in the oral cavity. The subsites of the oral cavity include the mucosal lip, buccal mucosa, alveolar ridge, retromolar trigone, floor of the mouth, hard palate, and oral tongue.^1^ Its peak incidence is between the sixth and seventh decades of life.^2^^,^^3^ Based on epidemiological data collected from the Brazilian National Cancer Institute (INCA),^4^ approximately 15,100 new cases of oral cavity neoplasms were reported in Brazil between 2023 and 2025. This represents the fifth most common form of malignancy in men, with 10900 cases.^4^ In the southeast region of Brazil, where São Paulo is located, it is the fourth most common form of tumors in men, with 5.830 cases.^4^

The development of Oral Squamous Cell Carcinoma (OSCC) is associated with numerous factors, including age, race, sex, inherited or acquired mutations, alcohol consumption, smoking, Human Papillomavirus (HPV) infection, a diet poor in fruits and vegetables, poor oral hygiene, and exposure to ultraviolet light.^5^^,^^6^ A close relationship has been reported to exist between OSCC and the abusive consumption of alcohol and tobacco, which are the primary risk factors and are present in approximately 90% of these cases.^7^

Although the oral cavity is an easily accessible region for an efficient and good-quality physical examination, the high incidence of advanced oral cancer in Brazil and worldwide indicates that there is a lack in the detection and diagnosis in its early phases.^8^ It could be related to a poor knowledge about the symptoms of oral cancer, difficulty in accessing the health services, and the inability of physicians and dentists to recognize signs and symptoms associated with malignant lesions in early phases.

Surgery is the main treatment for OSCC. Early-stage (I and II) tumors are usually treated through surgery or radiation; however, for advanced-stage (III and IV) tumors, a multimodality treatment is indicated, surgical resection followed by adjuvant radiation with or without chemotherapy.^9^ However, these patients have a high risk of locoregional recurrence even after treatment.^10^ This study aimed to analyze prognostic factors associated with survival and recurrence in patients with Oral Cavity Squamous Cell Carcinoma (OCSCC) treated in São Paulo, Brazil. The investigation focused on evaluating the influence of clinical stage, treatment modality, age, and sex on overall and recurrence-free survival in a large cohort derived from a public cancer registry.

## Methods

This was a single-center retrospective observational cohort study with a quantitative design. Data were extracted from the database of the Oncocenter Foundation of São Paulo (FOSP) after querying for patients diagnosed with oral cavity Squamous Cell Carcinoma (SCC) between 2004 and 2014 in the State of São Paulo, Brazil. A survival analysis was performed with the dataset obtained. Anatomical regions affected by oral cavity cancer were classified based on the diagnosis site according to the International Classification of Diseases (ICD-10) for Oncology, third edition.^11^ Patients diagnosed with a histological type compatible with SCC in the following ICD categories were selected: C00, C02, C02.1, C02.2, C02.3, C02.8, C02.9, C03, C04, C05.0, C05.8, C05.9, C06, and C14.8. The information collected from the database consisted only of numerical data and did not include personal and individual data of the patients.

The following categorical variables were analyzed: sex; age, categorized into four groups (<49, 50–59, 60–69, and ≥70-years); clinical stage at diagnosis (early or advanced); type of treatment (surgical or non-surgical); tumor site (region of the oral cavity affected by the malignant neoplasm); death; general and local tumor recurrences; and distant metastasis. The first age group (<49-years) included a smaller number of cases and therefore was not subdivided into 10-year intervals to maintain statistical consistency and avoid sparse data across categories. Epidemiologically, Oral Cavity Squamous Cell Carcinoma (OCSCC) predominantly affects older adults, with incidence increasing markedly after the sixth decade of life. In this cohort, patients younger than 50-years represented a minority and exhibited distinct clinical characteristics, often related to behavioral or viral etiologies rather than cumulative tobacco and alcohol exposure. Thus, grouping all patients under 49-years into a single category ensured adequate sample size for analysis and preserved the interpretability of age as a prognostic factor.

Definitions of recurrence and metastasis followed the criteria proposed in the AJCC Cancer Staging Manual, 8th Edition, and the NCCN Clinical Practice Guidelines in Oncology (NCCN Guidelines®): Head and Neck Cancers, as well as definitions applied in recent cohort studies on oral cavity squamous cell carcinoma^12^, ^13^, ^14^, ^15^:

• Local recurrence was defined as the reappearance of tumor growth at the primary site after completion of treatment.
• Regional recurrence referred to the reappearance of disease in the cervical lymph nodes within the regional drainage area.
• General recurrence was defined as any form of tumor recurrence, including local, regional, or distant disease.
• Distant metastasis referred to the development of tumor deposits in remote organs or tissues, such as the lungs, liver, or bones, confirmed by imaging or histopathology.

The FOSP database does not contain a dedicated variable for regional recurrence, which prevented this outcome from being analyzed separately. In this dataset, “general recurrence” was the only field that captured any recurrence event, encompassing local, regional, and distant failures. Only “local recurrence” (recurrence at the primary tumor site) was available as an isolated variable. Consequently, despite the clinical relevance of regional and locoregional recurrences ‒ which are frequently reported as the most common patterns of failure in OSCC and have a strong impact on survival^14^^,^^15^ ‒ the structure of the FOSP registry did not allow these outcomes to be evaluated independently in this study.

After thorough analysis of the variables mentioned, due to the lack of data, some patients were excluded only from a specific group.

The continuous variables analyzed were age, rate of progression time until death, rate of general and local recurrences, and time elapsed between diagnosis and treatment initiation. We evaluated time to death events as censored data, too.

To assess factors associated with death, general recurrence, and local recurrence, the data obtained from categorical and continuous variables were presented as descriptive variables and subjected to univariable and multivariable Cox regression analyses.

The coefficients were interpreted using Hazard Ratios (HRs), and the significance level was set at 5%. To describe the relationship among the outcomes (death, general recurrence, and local recurrence) and the variables, a Chi-Square test was performed. Subsequently, Kaplan-Meier curves were constructed with the estimates of disease progression-free survival and comparisons with the Log-rank test. The time-to-event outcomes were defined as follows:

• Overall Survival (OS): time from the date of diagnosis to death from any cause or last follow-up.
• Disease-Free Survival (DFS): time from the date of treatment completion to the first documented recurrence (local, regional, or distant) or death from any cause.
• Recurrence-Free Survival (RFS): time from the date of treatment completion to any recurrence (local, regional, or distant).
• Local Recurrence-Free Survival (LRFS): time from the date of treatment completion to recurrence at the primary tumor site.

OS was calculated from diagnosis, while DFS, RFS, and LRFS were measured from treatment completion, ensuring consistent definitions across endpoints according to their clinical relevance.

For statistical analysis, the SAS software (SAS Institute, Inc., Cary, NC, USA) for Windows, version 9.4, was used.

This study was approved by the Local Human Research Ethics Committee of the institution (State University of Campinas - Unicamp), under opinion number 16566319.6.0000.5404 and Certificate of Presentation for Ethical Consideration, through the Plataforma Brasil system. As this was a retrospective study based on secondary data from institutional databases, the requirement for informed consent was formally waived and approved by the Ethics Committee. The study was conducted in accordance with the ethical principles of the Declaration of Helsinki and the Brazilian National Health Council Resolution no. 466/2012.

## Results

Of the 10,122 patients with OSCC identified in the FOSP database, the mean follow-up time was 39.37 ± 37.93 months. Patient age ranged from 11 to 104 years, with a mean age of 60.2 ± 12.6 years at diagnosis. Most cases occurred in individuals aged ≥60-years (49.27%), and there was a predominance of males (78.3%) compared with females (21.7%). The tongue was the most common primary site, followed by the floor of the mouth and other or unspecified oral cavity subsites. A total of 1,612 patients were excluded from specific analyses due to missing or incomplete information: 364 lacked data on clinical stage, and 1,248 either received treatment modalities not included in this analysis, did not undergo any proposed treatment, or had missing treatment records. Among the remaining cases, 61.97% were diagnosed at an advanced clinical stage (III–IV) and 38.03% at an early stage (I–II). Regarding treatment, 69.62% underwent surgical therapy, while 30.38% received non-surgical treatment, including radiotherapy, chemotherapy, or combined chemoradiotherapy. Most patients (83.84%) did not experience recurrence, whereas 16.16% developed some form of recurrence; among these, 46.56% died (Table 1).

**Table 1:** Clinical characteristics and demographics of the patients.

Variables		Number of cases	Frequency (%)
Sex	Male	7,929	78.33
	Female	2,193	21.67
Age group (years)	10–49	1,941	19.17
	50–59	3,194	31.56
	60–69	2,628	25.96
	>70	2,359	23.31
Site	Tongue	3,609	35.65
	Floor of mouth	1,877	18.55
	Other and unspecified parts of the oral cavity	1,905	18.82
	Lip, gingiva, palate, and contiguous oral sites	2,731	26.98
Clinical stage	Advanced stage: III–IV	6,047	61.97
	Early stage: I–II	3,711	38.03
Type of treatment	Surgical	6,178	69.62
	Non-surgical	2,696	30.38
General recurrence	No	8,486	83.84
	Yes	1,636	16.16
Local recurrence	No	9,100	89.90
	Yes	1,022	10.10
Distant metastases	No	10,014	98.93
	Yes	108	1.07
Death	No	5,409	53.44
	Yes	4,713	46.56

The mean interval between diagnosis and treatment initiation was 69.50 ± 99.31 days. The mean time from treatment completion to local recurrence, general recurrence, and death was 37.48 ± 37.32, 36.14 ± 36.93, and 39.37 ± 37.93-months, respectively.

Univariable analysis revealed significant associations between sex, clinical stage, and treatment type with overall survival. Patients diagnosed at an advanced stage had a 5.43-fold higher risk of death (HR = 5.430; 95% CI 5.005–5.891), while those receiving non-surgical treatment had a 3.68-fold higher risk compared to surgically treated patients (HR = 3.678; 95% CI 3.452–3.920). Male patients showed a 28% higher probability of death (HR = 1.280; 95% CI 1.202–1.364). Local tumor recurrence and distant metastasis increased mortality risk by 37.2% (HR = 1.374; 95% CI 1.283–1.475) and 41% (HR = 1.414; 95% CI 1.133–1.753), respectively (Table 2).

**Table 2:** Results of Cox regression analysis of clinical factors associated with overall survival.

Variable	Category	Univariate analysis			Multivariate analysis (n = 8658)		
		p-value	HR^a^	95% CI	p-value	HR^a^	95% CI
Sex	Male × Female	<0.0001	1.280	1.189–1.378			
Stage	Advanced × Early	<0.0001	5.430	5.005–5.891	<0.0001	4.253	3.883–4.659
Type of treatment	Non-surgical × Surgical	<0.0001	3.678	3.452–3.920	<0.0001	2.546	2.381–2.722
Distant metastasis	Yes × No	0.0020	1410	1.133–1.753	0.0182	1.305	1.046–1.629
Local recurrence	Yes × No	<0.0001	1.372	1.268–1.484	<0.0001	1.306	1.203–1.417
Age	Numerical variable	0.0555	1.002	1.000–1.005	<0.0001	1.008	1.005–1.011
Interval between diagnosis and treatment beginning	Numerical variable	0.0679	1.022	0.998–1.045	<0.0001	0.999	0.999–0.999

a HR, Hazard Ratio for death; 95% CI, 95% Confidence Interval.

In the multivariable analysis, advanced clinical stage and non-surgical treatment remained independently associated with mortality, with 4.25- and 2.54-fold higher risks of death, respectively (HR = 4.253; 95% CI 3.883–4.659; HR = 2.546; 95% CI 2.381–2.722). Local tumor recurrence or distant metastasis also increased the likelihood of death by approximately 30% (HR = 1.306; 95% CI 1.203–1.417) (Table 2).

Advanced clinical stage, non-surgical treatment, and male sex were significantly associated with general recurrence in univariable analysis, with 2.1-fold (HR = 2.109; 95% CI 1.901–2.341), 1.5-fold (HR = 1.517; 95% CI 1.355–1.699), and 1.18-fold (HR = 1.180; 95% CI 1.045–1.332) increased risks, respectively. In the multivariable model, advanced stage and non-surgical treatment remained independent predictors, with 2.02-fold (HR = 2.021; 95% CI 1.810–2.256) and 1.23-fold (HR = 1.228; 95% CI 1.085–1.373) higher risks of general recurrence, respectively (Table 3). Both factors also influenced local recurrence: in univariable analysis, HRs were 2.093 (95% CI 1.835–2.388) for advanced stage and 1.734 (95% CI 1.508–1.994) for non-surgical treatment; in multivariable analysis, these remained significant (HR = 1.948; 95% CI 1.685–2.240 and HR = 1.402; 95% CI 1.211–1.622, respectively) (Table 4).

**Table 3:** Results of Cox regression analysis of clinical factors associated with recurrence-free survival.

Variable	Category	Univariate analysis			Multivariate analysis (n = 8655)		
		p-value	HR^a^	95% CI	p-value	HR^a^	95% CI
Sex	Male × Female	0.0078	1.180	1.045–1.332			
Stage	Advanced × Early	<0.0001	2.109	1.901–2.341	<0.0001	2.021	1.810–2.256
Type of treatment	Non-surgical × Surgical	<0.0001	1.517	1.355–1.699	0.0009	1.228	1.085–1.373
Age	Numerical variable	0.0633	0.996	0.992–1.000			
Interval between diagnosis and treatment beginning	Numerical variable	0.8866	1.003	0.963–1.045			

a HR, Hazard Ratio for death; 95% CI, Confidence Interval.

**Table 4:** Results of Cox regression analysis of clinical factors associated with local tumor recurrence.

Variable	Category	Univariate analysis			Multivariate analysis (n = 8655)		
		p-value	HR^a^	95% CI	p-value	HR^a^	95% CI
Sex	Male × Female	0.1943	1.105	0.950–1.285			
Stage	Advanced × Early	<0.0001	2.093	1.835–2.388	<0.0001	1.948	1.685–2.240
Type of treatment	Non-surgical × Surgical	<0.0001	1.734	1.508–1.994	<0.0001	1.402	1.211–1.622
Distant metastasis	Yes × No	0.5226	1.181	0.709–1.967			
Age	Numerical variable	0.1088	0.996	0.991–1.001			
Interval between diagnosis and treatment beginning	Numerical variable	0.9486	0.998	0.947–1.053			

a HR, hazard ratio for death; 95% CI, confidence interval.

Similarly, stages III–IV, non-surgical therapy, and male sex were associated with higher risk of death after recurrence in univariable analysis ‒ 2.23-fold (HR = 2.231; 95% CI 1.892–2.631), 1.89-fold (HR = 1.886; 95% CI 1.612–2.206), and 1.25-fold (HR = 1.253; 95% CI 1.039–1.511) increases, respectively. In the multivariable analysis, advanced stage and non-surgical treatment remained significant independent predictors, increasing post-recurrence mortality by 89.3% (HR = 1.893; 95% CI 1.590–2.255) and 50.2% (HR = 1.502; 95% CI 1.274–1.769), respectively. Distant metastasis approximately doubled the risk of death after recurrence (HR = 2.078; 95% CI 1.199–3.602) (Table 5).

**Table 5:** Results of Cox regression analysis of clinical factors associated with disease-free survival.

Variable	Category	Univariate analysis			Multivariate analysis (n = 975)		
		p-value	HR^a^	95% CI	p-value	HR^a^	95% CI
Sex	Male × Female	0.0182	1.253	1.039–1.511			
Stage	Advanced × Initial	<0.0001	2.231	1.892–2.631	<0.0001	1.893	1.590–2.255
Type of treatment	Non-surgical × Surgical	<0.0001	1.886	1.612–2.206	<0.0001	1.502	1.274–1.769
Distant metastasis	Yes × No	0.0577	1.701	0.983–2.945	0.0077	2.078	1.199–3.602
Age	Numerical variable	0.0026	1.009	1.003–1.016	0.0025	1.010	1.004–1.016
Duration between diagnosis and treatment initiation	Numerical variable	1.0000	1.000	0.938–1.066			
Time until recurrence	Numerical variable	<0.0001	0.967	0.963–0.972	<0.0001	0.968	0.963–0.972
						(1.033)	(1.028–1.038)

a HR, Hazard Ratio for death; 95% CI, Confidence Interval.

**Fig. 1:**
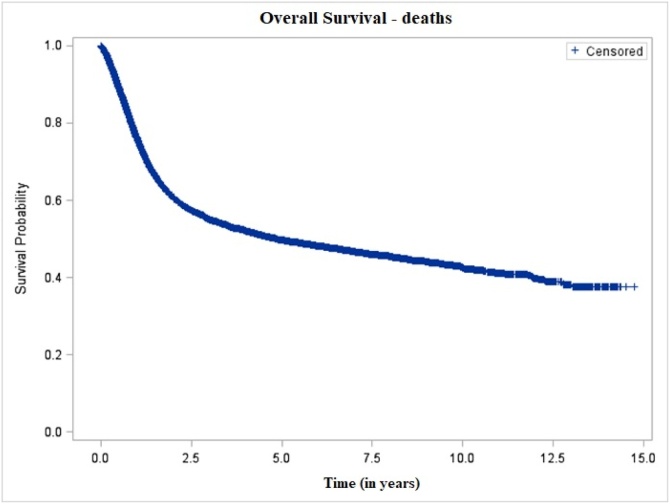
Overall survival of patients with oral cavity squamous cell carcinoma, São Paulo, 2004–2014.

**Fig. 2:**
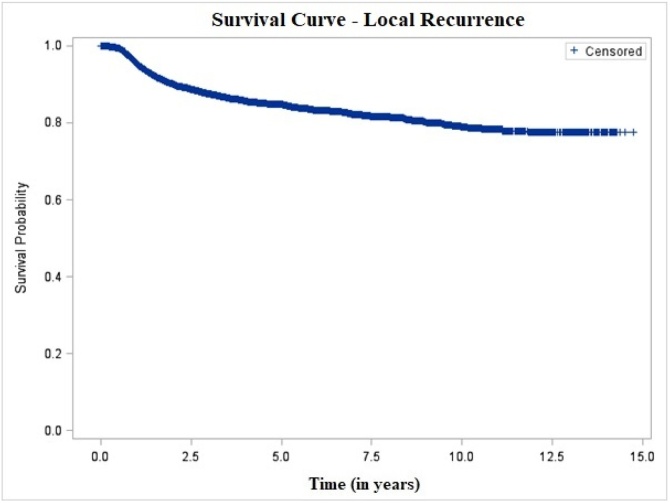
Local recurrence-free survival of patients with oral cavity squamous cell carcinoma, São Paulo, 2004–2014.

**Fig. 3:**
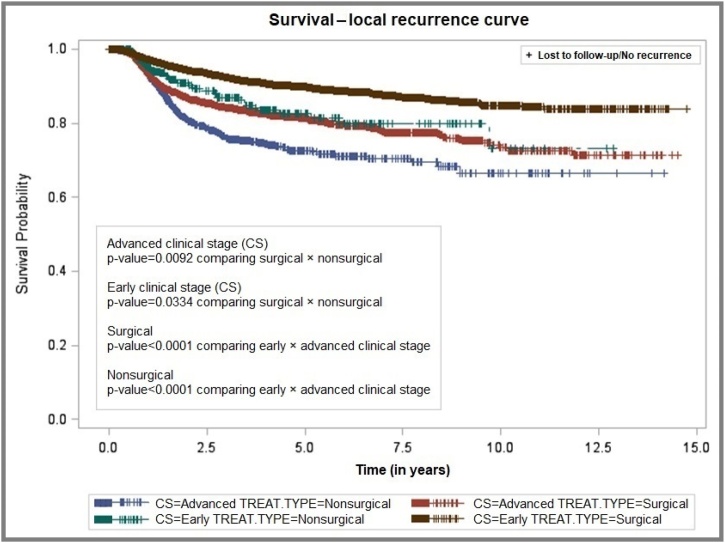
Local recurrence-free survival by clinical stage and treatment type, São Paulo, 2004–2014.

The 5-year and 10-year overall mortality probabilities were 50.37% and 57.62%, respectively, with a mean interval of 6.70 ± 0.06 years after diagnosis (Fig. 1). The probability of local recurrence was 15.27% at 5-years and 21.29% at 10-years, with a mean interval of 10.02 ± 0.05 years (Fig. 2). After stratification by clinical stage and treatment modality, patients with early-stage tumors who underwent surgery had the lowest local recurrence rates ‒ 10.17% and 15.69% at 5- and 10-years, respectively ‒ with a mean recurrence-free interval of 9.99 ± 0.06 years. In contrast, patients with advanced-stage tumors treated non-surgically showed the highest recurrence rates, reaching 24.97% and 33.46% at 5- and 10-years, with a shorter mean recurrence-free interval of 6.91 ± 0.12 years (Fig. 3).

**Fig. 4:**
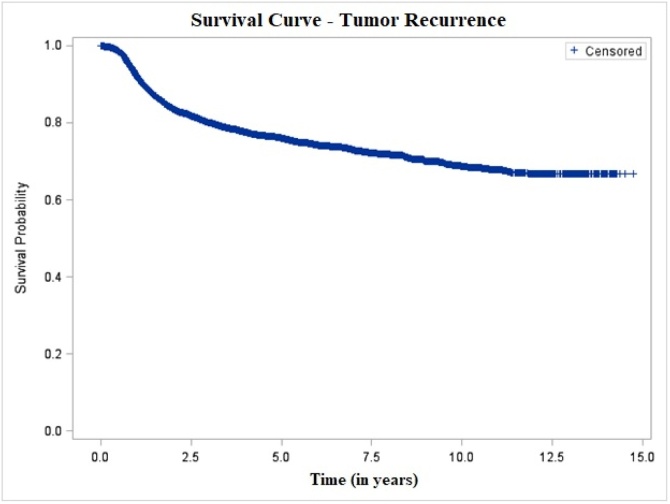
Tumor recurrence-free survival of patients with oral cavity squamous cell carcinoma, São Paulo, 2004–2014.

**Fig. 5:**
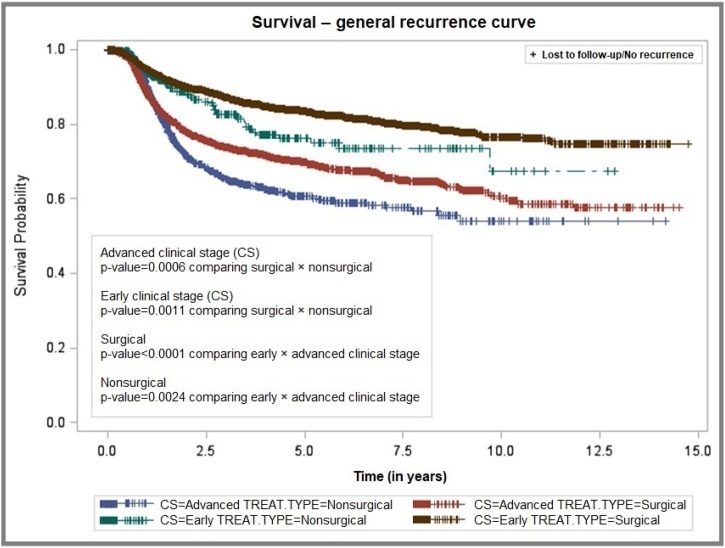
Overall recurrence-free survival by clinical stage and treatment type, São Paulo, 2004–2014.

The probability of general recurrence was 23.98% at 5-years and 31.54% at 10-years, with a mean recurrence-free interval of 9.07 ± 0.06 years (Fig. 4). Similar to the pattern observed for local recurrence, patients with early-stage tumors who underwent surgery demonstrated longer recurrence-free survival, with recurrence probabilities of 16.56% and 23.8% at 5- and 10-years, respectively, and a mean interval of 9.51 ± 0.08 years. Conversely, patients with advanced-stage tumors treated non-surgically exhibited higher recurrence rates ‒ 39.49% and 45.9% at 5- and 10-years ‒ with a shorter mean recurrence-free interval of 6.0 ± 0.13 years (Fig. 5).

## Discussion

This study, based on more than 10,000 patients with OSCC from a single-center public database, demonstrated that advanced clinical stage and non-surgical treatment were independently associated with worse survival and recurrence outcomes. A higher frequency of OSCC was observed among elderly patients, particularly those aged over 60-years (49.27%), and male sex was predominant (78.33%), which was also independently associated with increased risk of death and recurrence. Most patients (62%) were diagnosed at an advanced stage, directly influencing recurrence and survival rates. The mean interval from diagnosis to treatment initiation was 69-days. Surgical treatment, performed in 69% of cases, was associated with significantly improved overall and recurrence-free survival in both early and advanced stages, whereas non-surgical therapy was less effective in advanced disease. The overall 5-year survival rate was 50.37%, with a mean survival of 6.7-years after diagnosis, underscoring the critical prognostic role of stage at diagnosis and treatment modality.

Oral cavity cancer remains one of the most common malignancies worldwide, with a particularly high burden in developing countries, although incidence remains substantial in developed regions as well.^16^^,^^17^ According to Zhang et al.^18^ and Li et al.,^19^ approximately 660,000–890,000 new cases are diagnosed annually, resulting in 325,000–450,000 deaths. In Latin America, Brazil reports the highest incidence of oral cancer, particularly Squamous Cell Carcinomas (SCCs) of the oral cavity, lips, and oropharynx in São Paulo state.^20^^,^^21^

Brazilian studies indicate a higher prevalence of oral cancer among individuals older than 40-years, with peak incidence in the sixth and seventh decades of life.^21^^,^^22^ For example, Louredo et al.^21^ and Kowalski et al.^22^ reported mean ages of 60.3- and 61.1-years, respectively, consistent with data from other regions. In Japan, Jiromaru et al.^23^ reported a mean age of 69-years, while Sun et al.^24^ found 64.5-years in Australia, and Tenorio et al.^25^ observed 63.7-years for men and 64.1-years for women in Mexico. Compared to these cohorts, our study found a slightly higher mean age than reported in the United Arab Emirates (55.4-years)^26^ and Thailand (58.4-years),^27^ likely reflecting regional differences in demographics, healthcare access, and risk factor distribution.

Regarding sex distribution, the 2020 age-standardized incidence rates of oral cancer were 6.0 and 2.3 per 100,000 for men and women, respectively.^28^ In Japan, Jiromaru et al.^23^ reported a higher proportion among men (74.6%), and Al-Rawi et al.^26^ observed a male predominance of 71.4%, consistent with the well-established greater susceptibility of men to OSCC. Conversely, Dhanuthai et al.^27^ in Thailand reported 46.1% of cases in men and 53.8% in women, suggesting regional variations. Bai et al.^29^ also found that female patients in Beijing tended to be significantly older at diagnosis, indicating potential sex-related differences in onset and progression. Our findings confirm the predominance of male patients, consistent with global trends and local demographics.

This study also reinforces the persistent challenge of late-stage diagnosis in OSCC, with a substantial proportion of patients presenting with stage III or IV disease.^21^^,^^30^ Diagnostic delays often allow for local progression and regional metastasis.^30^ This pattern is observed both nationally and internationally. In Asia, Gajurel et al.^31^ reported that 49.7% of cases in Nepal were stage IV at diagnosis, while a 2024 clinicopathological series from Indonesia^32^ found 37.7% at this stage. Thompson-Harvey et al.^33^ reported a rising incidence of late-stage head and neck cancers in the United States, emphasizing delayed diagnosis as a global challenge and highlighting the need for public health initiatives promoting early detection. In Brazil, Pereira et al.^34^ observed that approximately 75% of patients with head and neck tumors, including oral cavity SCC, presented at advanced stages, reflecting disparities in healthcare access and substantial diagnostic delays within the public health system.

Beyond late diagnosis, treatment delays further contribute to worse outcomes. In our cohort, the mean interval between diagnosis and treatment initiation was longer than reported in other countries: in Australia, Kaing et al.^35^ found a mean of 30-days, while in Taiwan, Tsai et al.^36^ reported 24-days, with most patients initiating treatment within 30-days. Liao et al.^37^ demonstrated that longer diagnosis-to-treatment intervals were significantly associated with worse overall survival in OSCC.

Recent studies from China have shown that surgical treatment significantly improves both overall and recurrence-free survival in OSCC.^38^^,^^39^ Dong et al.^38^ reported 5-year overall and disease-free survival rates of 59.3% and 49.4%, respectively. Similarly, Ren et al.,^39^ in a prospective observational study of 174 patients with locally advanced OSCC treated exclusively with surgery, reported 5-year overall survival, disease-free survival, and locoregional control rates of 66.7%, 66.1%, and 82.4%, respectively. According to SEER Cancer Stat Facts (NCI, 2024), the combined 5-year relative survival rate for oral cavity and oropharyngeal cancers is approximately 68%,^40^ consistent with recent surgical series. In contrast, the lowest reported 5-year overall survival rate (20.7%) was observed in Uganda,^41^ reflecting significant disparities in healthcare access and treatment resources in low-income regions.

Considering that the oral cavity is easily accessible for physical examination, diagnostic delays in OSCC can be attributed to structural limitations of the Brazilian public health system (Unified Health System), including limited population coverage, insufficient professional training for early lesion detection, challenges in performing complementary exams, low public awareness of warning signs, and restricted access to specialized care.^42^ Consequently, delays in diagnosis and treatment initiation, often associated with advanced-stage presentation, have a major impact on prognosis, directly influencing mortality, recurrence, and overall survival.^43^^,^^44^ To contextualize our findings, a comparison with previously published OSCC series was performed (Table 6). The demographic characteristics and survival outcomes in our cohort are consistent with those reported in both national and international studies.

**Table 6:** Comparative summary of major OSCC series.

Region / Study (Year)	N	Mean Age (years)	Male (%)	Advanced Stage (III–IV) (%)	Primary Treatment	Diagnosis-to-treatment interval (days)	5-year OS (%)	5-year DFS (%)
South America								
Current Study, Brazil (2004–2014)	10,518	61	78.3	62	Surgery 69%	69	50.4	–
Louredo et al., Brazil (2022)	–	60.3	–	–	–	–	–	–
Kowalski et al., Brazil (2020)	–	61.1	–	–	–	–	–	–
Tenorio et al., Mexico (2024)	–	63.7 (M), 64.1 (F)	–	–	–	–	–	–
Asia								
Jiromaru et al., Japan (2024)	–	69	74.6	–	–	–	–	–
Al-Rawi et al., UAE (2023)	–	55.4	71.4	–	–	–	–	–
Dhanuthai et al., Thailand (2018)	–	58.4	46.1	–	–	–	–	–
Dong et al., China (2024)	174	–	–	–	Surgery only	–	59.3	49.4
Ren et al., China (2024)	174	–	–	–	Surgery only	–	66.7	66.1
Oceania								
Sun et al., Australia (2023)	–	64.5	–	–	–	30	–	–
North America								
SEER (USA, 2024)	–	–	–	–	Mixed	–	68	–
Africa								
Asio et al., Uganda (2018)	–	–	–	–	Mixed	–	20.7	–

Notes: OS, Overall Survival; DFS, Disease-Free Survival.

Advanced stage defined as AJCC III–IV.

Data not reported in original publications are indicated as “–”.

Diagnosis-to-treatment interval reflects time from confirmed diagnosis to treatment initiation.

This study has several limitations that should be considered. First, because it relied on a retrospective public database maintained by FOSP, it was not possible to determine the specific causes of death, nor to access important clinical and histopathological variables such as smoking and alcohol consumption, surgical margin status, number of positive lymph nodes, extracapsular spread, perineural invasion, depth of invasion, and comorbidities. The database structure also restricted the analysis of recurrence patterns: although local and general recurrence were available, regional and locoregional recurrences ‒ recognized in the literature as the most frequent and prognostically significant failure patterns^14^^,^^15^ ‒ could not be evaluated separately, limiting a more detailed interpretation of disease behavior. In addition, the dataset encompasses diagnoses made between 2004 and 2014, which may not fully reflect current diagnostic pathways, treatment protocols, or improvements in healthcare access. Future research should incorporate prospective designs, molecular and pathological biomarkers, detailed recurrence mapping, and evaluations of early-detection and multimodal treatment strategies to refine prognostic assessment and improve outcomes for patients with OSCC.

## Conclusion

This population-based study, including over 10,000 patients with oral cavity squamous cell carcinoma (OSCC) in São Paulo, Brazil, identified clinical stage and treatment modality as the most significant independent prognostic factors for survival and recurrence. Advanced-stage patients (III–IV) and those receiving non-surgical therapy exhibited substantially reduced overall and recurrence-free survival. Age and sex also showed prognostic relevance, with older and male patients at higher risk of recurrence and mortality.

These findings reinforce the importance of early diagnosis and timely surgical intervention as key determinants of improved outcomes in OSCC. Moreover, they highlight the need for public health strategies aimed at reducing diagnostic and treatment delays, particularly within the public healthcare system, where most cases are concentrated. Strengthening awareness, screening, and referral pathways could contribute substantially to enhancing survival rates and reducing the disease burden associated with oral cancer in Brazil. Future studies incorporating molecular prognostic biomarkers and population-based surveillance are warranted to refine risk stratification and guide more effective interventions.

## ORCID ID

Isabella Grieger: 0000-0002-7094-0378

Daniel Naves de Araújo: 0000‑0002‑2447‑9664

## CRediT authorship contribution statement

Gustavo Mercuri: Data preparation; data compilation and analysis; paper writing; final manuscript review and approval.

Isabella Grieger: Data preparation; data compilation, and analysis.

Daniel Naves de Araújo: Data compilation and analysis.

Fabio Lau: Data compilation and analysis.

Carlos Takahiro Chone: Study design; data preparation, data analysis; final manuscript review and approval.

## Data availability statement

The authors declare that all data are available in repository.

## Declaration of competing interest

The authors declare no conflicts of interest.
